# Screening for post-traumatic stress disorder after injury in the pediatric emergency department - a systematic review protocol

**DOI:** 10.1186/2046-4053-3-19

**Published:** 2014-03-02

**Authors:** Jeffrey Odenbach, Amanda Newton, Rebecca Gokiert, Cathy Falconer, Craig Courchesne, Sandra Campbell, Sarah J Curtis

**Affiliations:** 1 Department of Pediatrics, University of Alberta, Edmonton Clinic Health Academy, 11405-87 Avenue, Edmonton, AB T6G 1C9, Canada; 2 Women & Children’s Health Research Institute, University of Alberta, 4-081 Edmonton Clinic Health Academy (ECHA)11405, 87 Avenue, Edmonton, AB T6G 1C9, Canada; 3 Faculty of Extension, University of Alberta, 2-281 Enterprise Square, Edmonton, AB T5J 4P6, Canada; 4 Stollery Children’s Hospital, 8440-112 Street, Edmonton, AB T6G 2B7, Canada; 5 John W. Scott Health Sciences Library, 2 K3.28 Walter C. Mackenzie Health Sciences Centre, University of Alberta, Edmonton, AB T6G 2R7, Canada

**Keywords:** Post-traumatic stress disorder (PTSD), Screening, Instrument, Emergency department, Pediatrics, Injury

## Abstract

**Background:**

Pediatric injury is highly prevalent and has significant impact both physically and emotionally. The majority of pediatric injuries are treated in emergency departments (EDs), where treatment of physical injuries is the main focus. In addition to physical trauma, children often experience significant psychological trauma, and the development of acute stress disorder (ASD) and post-traumatic stress disorder (PTSD) is common. The consequences of failing to recognize and treat children with ASD and PTSD are significant and extend into adulthood. Currently, screening guidelines to identify children at risk for developing these stress disorders are not evident in the pediatric emergency setting. The goal of this systematic review is to summarize evidence on the psychometric properties, diagnostic accuracy, and clinical utility of screening tools that identify or predict PTSD secondary to physical injury in children. Specific research objectives are to: (1) identify, describe, and critically evaluate instruments available to screen for PTSD in children; (2) review and synthesize the test-performance characteristics of these tools; and (3) describe the clinical utility of these tools with focus on ED suitability.

**Methods:**

Computerized databases including MEDLINE, EMBASE, CINAHL, ISI Web of Science and PsycINFO will be searched in addition to conference proceedings, textbooks, and contact with experts. Search terms will include MeSH headings (post-traumatic stress or acute stress), (pediatric or children) and diagnosis. All articles will be screened by title/abstract and articles identified as potentially relevant will be retrieved in full text and assessed by two independent reviewers. Quality assessment will be determined using the QUADAS-2 tool. Screening tool characteristics, including type of instrument, number of items, administration time and training administrators level, will be extracted as well as gold standard diagnostic reference properties and any quantitative diagnostic data (specificity, positive and negative likelihood/odds ratios) where appropriate.

**Discussion:**

Identifying screening tools to recognize children at risk of developing stress disorders following trauma is essential in guiding early treatment and minimizing long-term sequelae of childhood stress disorders. This review aims to identify such screening tools in efforts to improve routine stress disorder screening in the pediatric ED setting.

**Trials registration:**

PROSPERO registration: CRD42013004893

## Background

Pediatric injury is highly prevalent and has significant impact. Injuries are the largest cause of morbidity and mortality among children in the United States and Canada, and are the leading cause of emergency department (ED) visits for children greater than 1 year of age [1-3]. Injury-related ED visits in the United States are estimated to range from 63 to 165 per 1,000 children [4]. The vast majority of pediatric injuries are treated acutely in EDs with a strong focus on physical trauma.

The prevalence of stress disorders such as acute stress disorder (ASD) and post-traumatic stress disorder (PTSD) following injury is high. Some children with sub-clinical features suffer as much distress and impairment as those diagnosed with full PTSD, and the risk factors to identify those children at greatest risk for subsequent development of PTSD have been outlined [5]. Recent research has demonstrated that up to 68% of all children experience potentially traumatic events, with more than half of these children experiencing multiple events [6]. A recent systematic review of 21 single-center cohort studies reported the prevalence of PTSD among pediatric survivors of motor vehicle accidents (MVA) to be between 12% and 46% at 4 months post-MVA and 13 to 25% at 12 months post-MVA [7]. A prospective cohort study of children experiencing orthopedic trauma including bone fractures secondary to falls, sporting injuries or MVAs found that 33% of the cohort experienced PTSD at 1 year follow-up [8]. Historically it was thought that PTSD symptoms would only develop after experiencing trauma resulting in severe injury. It has recently been discovered, through cohort studies in children, that these considerably high rates of ASD and PTSD do not seem to depend on the nature or the severity of the injury sustained [7] and even mild trauma can have long-lasting effects [8-10]. Explanations for this are variable, multifactorial and complex, but biological plausibility for this effect is present [11]. Some individuals may be identifiably at higher risk for the development of subsequent stress disorders because of key pre-traumatic, peri-traumatic, and post-traumatic factors that are individually specific but that over a population are identifiable.

The consequences of failing to recognize and treat children at risk for PTSD are stark and prevail into adult life. PTSD is linked to changes in neurobiology [12,13] during development and highly correlated with poor quality of life [14]. Long-term consequences of unrecognized and untreated ASD and PTSD include increased lifetime risk of poor school performance, impaired development, aggression, risk-taking behaviors, depression, suicidal ideation, increased adult mental illness and increased adult physical illness [15-19].

The ED is usually the first and is often the only point of medical care for children experiencing a physical trauma. Thus, the ED visit may be the best opportunity to screen for PTSD vulnerability and to connect patients to resources for future interventions or treatments [20,21]. Such screening, however, is not current practice in this setting. In 2008, the American Academy of Pediatrics (AAP) released a position statement on the management of pediatric trauma stating that psychological support is a vital component of pediatric trauma care [22]. The AAP has also recently released a report describing the importance of the role of ED healthcare professionals in the stabilization and discovery of pediatric mental health, and advocating for improved recognition and treatment of mental health needs [23]. Despite these strong recommendations, psychological support as a component of follow-up for pediatric trauma is lacking or absent [24,25]. Reasons for this are variable, but time pressures and lack of awareness of appropriate screening tools are key contributors. This review aims to address this deficiency by conducting a comprehensive review in order to identify PTSD screening tools that are suitable for use in the pediatric ED setting. Specific research objectives are to: (1) identify, describe, and critically evaluate instruments available to screen for PTSD in children and adolescents; (2) review and synthesize the test-performance characteristics of these tools; and (3) describe the clinical utility of these tools with particular focus on suitability for ED use. A broad systematic synthesis of the evidence regarding the availability and performance of available tools applicable to this very important setting does not yet exist.

## Methods

The systematic review protocol has been registered with the PROSPERO systematic review database (registration number CRD42013004893) and has been designed based on the PRISMA statement and guidelines established in the *Cochrane Handbook for Systematic Reviews of Interventions* Version 5.1.0.

### Search strategy/data sources

Database searches will be executed by a medical librarian (SC) using subject headings (for example, MESH, EMTREE) and text words to retrieve articles related to the following concepts: (post-traumatic stress or acute stress) and (pediatric or children) and diagnosis. Databases searched will include: MEDLINE, EMBASE, EBM Reviews (Cochrane database of systematic reviews, ACP journal club, Database of abstracts of reviews of effects, Cochrane central register of controlled trials, Cochrane methodology register, Health technology assessment, NHS economic evaluation database), Global Health, PsycINFO, Health and Psychosocial Instruments, ProQuest dissertations and theses as well as conference proceedings and contact with experts in the field. An additional search of gray literature will include Google Scholar and related field websites in pediatric mental health with screening tools evaluating PTSD in children. There will be no restrictions for articles by language, publication date or publication status. All references will be exported to RefWorks citation management software, where duplicates will be verified, recorded and removed. Search strategies are listed in Additional file 1: Appendix 3.

### Study selection

Two independent reviewers will screen all articles by title and abstract against inclusion and exclusion criteria described in Table 1. Any discrepancies between the two reviewers throughout the screening/selection process will be resolved by discussion and consultation with a third reviewer. Articles identified as potentially relevant will be retrieved for full text review and all non-English articles will be translated appropriately. Detailed eligibility forms (Additional file 1: Appendix 1) will be completed for all potentially relevant articles to confirm eligibility criteria and record details of excluded articles.

**Table 1:** Inclusion/exclusion criteria

**Inclusion criteria**	**Exclusion criteria**
Articles identifying, describing or evaluating one or more ASD/PTSD screening or diagnostic tools.	Articles that do not identify, describe or evaluate an ASD/PTSD screening or diagnostic tool
Articles focusing on ASD/PTSD/stress disorders	Broad articles on multiple mental health issues/psychiatric conditions
Articles focusing on children and adolescents under 18 years of age	Articles including adults aged 18+ or articles where age is not provided
Adequate follow-up of at least 1 month described	Follow-up period undefined or less than 1 month
Articles with a described mechanism of injury not listed in the exclusion criteria	Articles without a described mechanism of injury or articles describing trauma resulting from warfare, terrorism, abuse, neglect, medical illness or natural disaster
Systematic reviews, descriptive studies, cohort studies, case series, randomized and quasi-randomized trials	Narrative review articles, commentaries or letters to the editor

ASD, acute stress disorder; PTSD, post-traumatic stress disorder.

### Types of interventions, participants, and study designs

Screening tools, instruments, or questionnaires implemented after traumatic events with the purpose of identifying children/adolescents (<18 years) at risk for developing PTSD are eligible. Such measures, with diagnostic and psychometric study designs, will be described and evaluated. Studies will clearly describe the gold-standard criteria by which a diagnosis of PTSD was made (diagnostic studies) [26] or comparison instruments (psychometric studies) [27].

### Data extraction and analysis

Two reviewers will utilize a customized data extraction form (Additional file 1: Appendix 2) based on a modified version of the Social-Emotional Tool described by Gokiert and colleagues [28]. Across all studies, screening tool characteristics will be extracted and described (for example, types of questions, length) as will characteristics about the population used to develop and evaluate the intervention and the setting in which it was evaluated. Where psychometric data are provided, reliability and validity data will be extracted and summarized. Where diagnostic data are provided, the following will be calculated: (1) sensitivity (with 95% CIs), (2) specificity (with 95% CIs), (3) likelihood ratios (with 95% CIs), (4) diagnostic odds ratios (with 95% CIs), and (5) receiver operating characteristic curves. To assess potential discrepancy between differences in the reliability of the diagnostic gold standard used between studies, subgroup analysis will be preformed according to the QUADAS-2 tool [26] for assessment of risk of bias and applicability.

### Quality assessment

Review of citations/abstracts, included articles, data extraction, and quality rating will be performed by two team members using customized forms with pre-determined criteria (Additional file 1: Appendices 1 and 2); inter-rater reliability (kappa statistic) will be measured. Where possible, diagnostic data will be arranged in 2 × 2 tables to calculate the above outcome measures and pooled to produce summary likelihood ratios for positive and negative test results. Quality of included studies will be determined using the QUADAS-2 tool for assessment of diagnostic accuracy in systematic reviews. Heterogeneity will be explored and subgrouping, meta-regression, or graphical methods will be employed if warranted. The degree of heterogeneity via I2 statistic will be calculated when applicable. If substantial heterogeneity exists (I2 > 25%), subgrouping by demographics (age, gender, location) and meta-regression analysis will be attempted.

## Discussion

The ED is a unique environment. The pace, range of conditions and stress is high. Healthcare providers have multiple competing care duties and the chances of successfully implementing a screening tool into clinical care pathways for pediatric traumatic events will be maximized if the tool is carefully selected to suit the environment while maintaining accuracy in diagnosis to ensure improved outcomes. Given that pediatric injury is so prevalent and that EDs support the vast majority of these injuries acutely, it is logical for clinicians familiar with this unique environment and its patients to review and select the most suitable tool possible. To date, we are unaware of the availability of any such comprehensive review pertaining to the use of such tools in the acute-care ED. This review will address this deficiency by identifying, describing and evaluating such tools and comparing their features in a concise table. We will make screening recommendations based on the tools described and provide a foundation for the development of future practice guidelines in the psychological management of pediatric injury.

## Abbreviations

AAP: American Academy of Pediatrics; ASD: acute stress disorder; ED: emergency department; MVA: motor vehicle accidents; PTSD: post-traumatic stress disorder.

## Competing interests

The authors declare that they have no competing interests.

## Authors’ contributions

JO, the project lead, will complete PROSPERO registration, compile search results, remove duplicates, participate in screening, article retrieval, quality assessment and manuscript drafting. CC will participate in article screening, retrieval and quality assessment as well as provide revisions to the final manuscript. SC will develop and execute database search protocol and draft the database search manuscript methods section. SJC, AN, RG and CF initiated the project concept, will provide experience in evidence-based medicine methodology and data interpretation as well as contribute in manuscript preparation and review. SJC also drafted the initial review protocol and will act as third reviewer for article screening and inclusion. All authors read and approved the final manuscript.

## Supplementary Material

Additional file 1 **Appendix 1.** Eligibility form for potentially relevant articles. **Appendix 2.** Data extraction form. **Appendix 3.** Search Strategy. Click here for file
