# Supplementary material for: Screening for post-traumatic stress disorder after injury in the pediatric emergency department - a systematic review protocol
Source: Syst Rev. 2014 Mar 2;3:19. doi: 10.1186/2046-4053-3-19 (PMC3944964; doi:10.1186/2046-4053-3-19)
Supplement: Additional file 1 — Appendix 1. Eligibility form for potentially relevant articles. Appendix 2. Data extraction form. Appendix 3. Search Strategy. [file 2046-4053-3-19-S1.doc]

**Appendix 1. Eligibility form for potentially relevant articles**

| Title:  Authors:  Journal:  Year:  Page: |  |
| --- | --- |

| **Type of study**: |  |
| --- | --- |
| **Must** identify, describe or evaluate / assess at least one ASD / PTSD screening tool: | Yes / No |
| **Must** be a Pediatric Study (0-18 years)? | Yes / No |
| **Must** describe follow-up at least 1 month: | Yes / No |
| **Must** describe mechanism of injury**: | Yes / No |
| Include the study in the review? | Yes / No |
| Do both reviewers agree?  Why was the study excluded? What are concerns? | Yes / No |
| Third reviewer decision | Include/Exclude |
| Were the authors contacted?  Published data? | Yes/No  Yes/ No |

*Mechanism of injury/trauma cannot be a result of: warfare, terrorism, abuse, neglect, medical illness or natural disaster.

Comments:___________________________________________________________________________________________________________________________________________________________________________________________________________________

________________________________________________________________________________________________________________

Reminder of exclusion criteria:

- Articles that do not identify, describe or evaluate at least one ASD / PTSD screening tool.
- Articles including adults (18+) or that do not specify age.
- Follow-up period unlisted or <1 month.
- Articles without a described mechanism of injury or injury/trauma resulting from any of the following: warfare, terrorism, abuse, neglect, medical illness or natural disaster.

**Appendix 2. Data extraction form**

| Title:  Authors:  Journal:  Year:  Page: |
| --- |

| Type of study: |  |
| --- | --- |
| Name of screening tool used: |  |
| Type of screening tool: (survey, questionnaire, checklist etc.) |  |
| # of items / administration time: |  |
| Training level required to administer: |  |
| Mechanism of injury/trauma: |  |
| Treatment / care setting: |  |
| Patients included (# total): |  |
| Age (mean, median, range): |  |
| Gender (#, % Male/Female): |  |
| Duration of follow-up (months): |  |
| Patients screened (#, %)  Diagnosed with PTSD (#, %)  Not meeting diagnostic criteria (#, %) |  |
| Control patients not screened (#, %)  Diagnosed with PTSD (#, %)  Not meeting diagnostic criteria (#, %) |  |
| Sensitivity / Specificity (%): |  |
| Positive / negative likelihood ratios: |  |
| Positive / negative odds ratios: |  |
| Interrater reliability: |  |
| Test-retest reliability: |  |
| Internal consistency: |  |
| Name of reference (gold standard) diagnostic test: |  |
| Type of reference (gold standard) diagnostic test: (semi-)structured interview, checklist, self-report etc. |  |
| Level of training of reference (gold standard) diagnostic test administrator: |  |
| Time between traumatic experience / initial screening and reference (gold standard) diagnostic test: |  |
| QUADAS-2 risk of bias: (low, medium. high) |  |
| Length appropriate for ED use? |  |
| Tool concise enough for ED use? |  |
| Language of tool age appropriate? |  |

**Appendix 3: PTSD Search Strategy**

Ovid Technologies, Inc.
------------------------------
Database: Ovid MEDLINE(R) In-Process & Other Non-Indexed Citations, Ovid MEDLINE(R) Daily and Ovid MEDLINE(R) <1946 to Present> Searched June 13, 2013
Search Strategy:
--------------------------------------------------------------------------------
1     [ptsd.mp](http://ptsd.mp/). [mp=title, abstract, original title, name of substance word, subject heading word, keyword heading word,
protocol supplementary concept, rare disease supplementary concept, unique identifier] (11512)
2     post traumatic [stress.mp](http://stress.mp/). [mp=title, abstract, original title, name of substance word, subject heading word, keyword heading word, protocol supplementary concept, rare disease supplementary concept, unique identifier] (5990)
3     (acute stress disorder or acute stress symptom*).mp. [mp=title, abstract, original title, name of substance word, subject heading word, keyword heading word, protocol supplementary concept, rare disease supplementary concept, unique identifier] (419)
4     neuroses [posttraumatic.mp](http://posttraumatic.mp/). or exp *Stress Disorders, Post-Traumatic/ or *"Stress, Psychological"/di [Diagnosis] (17323)
5     (stress adj1 posttraumatic).mp. [mp=title, abstract, original title, name of substance word, subject heading word, keyword heading word, protocol supplementary concept, rare disease supplementary concept, unique identifier] (10665)
6     1 or 2 or 3 or 4 or 5 (23907)
7     (pediatric$ or paediatric$ or child$ or adolescen$ or preschool$ or pre-school$ or teen$ or kindergarten* or kindergarden$ or elementary school$ or nursery school$ or youth$ or schoolchild$ or toddler$ or boy or boys or girl* or pubescen* or pre-pubesc*).mp. or (child* or adolesc* or pediat* or paediat*).jn. (2780944)
8     exp Child/ (1511774)
9     Pediatrics/ (38862)
10     adolescent/ or child, abandoned/ or child, exceptional/ or "child of impaired parents"/ or child, orphaned/ or child, unwanted/ or minors/ (1573147)
11     7 or 8 or 9 or 10 (2781274)
12     diagnostic tool*.mp. (24089)
13     screening test*.mp. (22140)
14     test instrument*.mp. (396)
15     screening.ti. (99064)
16     "Questionnaires"/ (286997)
17     "Checklist"/ (1787)
18     "Psychometrics"/ (52927)
19     "Psychiatric Status Rating Scales"/ (57982)
20     12 or 13 or 14 or 15 or 16 or 17 or 18 or 19 (501149)
21     6 and 11 and 20 (2810)

Ovid Technologies, Inc.
------------------------------


Database: Embase <1974 to 2013 June 18>
Search Strategy:
--------------------------------------------------------------------------------
1     [ptsd.mp](http://ptsd.mp/). or exp posttraumatic stress disorder/ (32501)
2     ((post traumatic or posttraumatic) adj2 (stress* or psychos?s or psychic or disorder* or syndrome*or
neuros?s)).ti,ab. [mp=title, abstract, subject headings, heading word, drug trade name, original title, device
manufacturer, drug manufacturer, device trade name, keyword] (19830)
3     *acute stress/ or *acute stress disorder/ (977)
4     acute stress*.ti,ab. (5986)
5     1 or 2 or 3 or 4 (39664)
6     *child/ (95866)
7     *adolescent/ (28198)
8     (pediatric* or paediatric* or child or children* or adolescen* or preschool* or pre-school* or teen$ or
kindergarden$ or kindergarten* or elementary school$ or nursery school$ or youth$ or schoolchild* or toddler$ or boy or
boys or girl* or pubescen* or juvenile* or adolesc*or pre-pubesc*).mp. or (child or children or adolesc* or pediat* or
paediat*).jn. (2801936)
9     exp *pediatrics/ (44485)
10     6 or 7 or 8 or 9 (2803847)
11     (named inventories questionnaires and rating scales).mp. [mp=title, abstract, subject headings, heading word,
drug trade name, original title, device manufacturer, drug manufacturer, device trade name, keyword] (5189)
12     (diagnos* adj1 (method* or test* or tool*)).mp. [mp=title, abstract, subject headings, heading word, drug trade
name, original title, device manufacturer, drug manufacturer, device trade name, keyword] (331146)
13     exp Child Behavior Checklist/ or "Posttraumatic Diagnostic Scale"/ or checklist*.mp. or exp checklist/ (25506)
14     exp questionnaire/ or exp Childhood Trauma Questionnaire/ or questionnaire*.mp. (484228)
15     exp clinical assessment tool/ or clinical assessment tool*.mp. (12308)
16     exp screening/ or exp psychologic test/ or psychological [screening.mp](http://screening.mp/). or exp psychological aspect/ (1065482)
17     11 or 12 or 13 or 14 or 15 or 16 (1769926)
18     5 and 10 and 17 (4318)

Ovid Technologies, Inc.
------------------------------
Database: EBM Reviews - Cochrane Database of Systematic Reviews <2005 to April 2013>, EBM Reviews - ACP Journal Club <1991 to May 2013>, EBM Reviews - Database of Abstracts of Reviews of Effects <2nd Quarter 2013>, EBM Reviews – Cochrane Central Register of Controlled Trials <May 2013>, EBM Reviews - Cochrane Methodology Register <3rd Quarter 2012>, EBM Reviews - Health Technology Assessment <2nd Quarter 2013>, EBM Reviews - NHS Economic Evaluation Database <2nd Quarter 2013> Searched June 13, 2013

Search Strategy:
--------------------------------------------------------------------------------
1     [ptsd.mp](http://ptsd.mp/). [mp=ti, ab, tx, kw, ct, ot, sh, hw] (990)
2     post traumatic [stress.mp](http://stress.mp/). [mp=ti, ab, tx, kw, ct, ot, sh, hw] (489)
3     (acute stress disorder or acute stress symptom*).mp. [mp=ti, ab, tx, kw, ct, ot, sh, hw] (52)
4     neuroses [posttraumatic.mp](http://posttraumatic.mp/). or exp *Stress Disorders, Post-Traumatic/ or *"Stress, Psychological"/di [Diagnosis]
(47)
5     (stress adj1 posttraumatic).mp. [mp=ti, ab, tx, kw, ct, ot, sh, hw] (762)
6     1 or 2 or 3 or 4 or 5 (1540)
7     (pediatric$ or paediatric$ or child$ or adolescen$ or preschool$ or pre-school$ or teen$ or kindergarten* or
kindergarden$ or elementary school$ or nursery school$ or youth$ or schoolchild$ or toddler$ or boy or boys or girl* or pubescen* or pre-pubesc*).mp. or (child* or adolesc* or pediat* or paediat*).jn. (133613)
8     exp Child/ (38737)
9     Pediatrics/ (406)
10     adolescent/ or child, abandoned/ or child, exceptional/ or "child of impaired parents"/ or child, orphaned/ or
child, unwanted/ or minors/ (71806)
11     7 or 8 or 9 or 10 (133613)
12     diagnostic tool*.mp. (545)
13     screening test*.mp. (1728)
14     test instrument*.mp. (59)
15     screening.ti. (5233)
16     "Questionnaires"/ (14042)
17     "Checklist"/ (31)
18     "Psychometrics"/ (2089)
19     "Psychiatric Status Rating Scales"/ (6987)
20     12 or 13 or 14 or 15 or 16 or 17 or 18 or 19 (28265)
21     6 and 11 and 20 (90)

Ovid Technologies, Inc.
------------------------------
Database: Global Health <1910 to 2013 Week 22> Searched June 13, 2013
Search Strategy:
--------------------------------------------------------------------------------
1     [ptsd.mp](http://ptsd.mp/). [mp=abstract, title, original title, broad terms, heading words] (771)
2     post traumatic [stress.mp](http://stress.mp/). [mp=abstract, title, original title, broad terms, heading words] (984)
3     (acute stress disorder* or acute psychological stress or acute stress symptom*).mp. [mp=abstract, title, original
title, broad terms, heading words] (30)
4     (stress adj1 posttraumatic).mp. [mp=abstract, title, original title, broad terms, heading words] (706)
5     "post-traumatic stress disorder".sh. (600)
6     1 or 2 or 3 or 4 or 5 (1338)
7     "children".sh. or (pediatric$ or paediatric$ or child$ or adolescen$ or preschool$ or pre-school$ or teen$ or kindergarten* or kindergarden$ or elementary school$ or nursery school$ or youth$ or schoolchild$ or toddler$ or boy or boys or girl* or pubescen* or pre-pubesc*).mp. or (child* or adolesc* or pediat* or paediat*).jn. (315970)
8     adolescent/ or child, abandoned/ or child, exceptional/ or "child of impaired parents"/ or child, orphaned/ or child, unwanted/ or minors/ (32307)
9     7 or 8 (315970)
10     diagnostic tool*.mp. or "diagnostic techniques".sh. (37871)
11     screening test*.mp. or "screening".su. (34471)
12     test instrument*.mp. (29)
13     screening.ti. (21938)
14     "Questionnaires"/ or questionnaire*.mp. (69407)
15     "Checklist"/ or checklist*.mp. (3059)
16     10 or 11 or 12 or 13 or 14 or 15 (143019)
17     6 and 9 and 16 (101)

Ovid Technologies, Inc.
------------------------------
Database: PsycINFO <1987 to June Week 2 2013>
Search Strategy:
--------------------------------------------------------------------------------
1     [ptsd.mp](http://ptsd.mp/). [mp=title, abstract, heading word, table of contents, key concepts, original title, tests & measures]
(19590)
2     post traumatic [stress.mp](http://stress.mp/). [mp=title, abstract, heading word, table of contents, key concepts, original title,
tests & measures] (6718)
3     (acute stress disorder or acute stress symptom*).mp. [mp=title, abstract, heading word, table of contents, key
concepts, original title, tests & measures] (707)
4     stress disorders, traumatic/ or stress disorders, post-traumatic/ or stress disorders, traumatic, acute/ (0)
5     acute [stress.mp](http://stress.mp/). (2505)
6     1 or 2 or 3 or 4 or 5 (23872)
7     pediatric*.mp. (23179)
8     (pediatric$ or paediatric$ or child$ or adolescen$ or preschool$ or pre-school$ or teen$ or kindergarden$ or
kindergarten* or elementary school$ or nursery school$ or youth$ or schoolchild$ or toddler$ or boy or boys or girl* or
pubescen* or pre-pubesc*).mp. or (child* or adolesc* or pediat* or paediat*).jn. (520156)
9     exp Pediatrics/ (12738)
10     7 or 8 or 9 (520156)
11     *"Test Validity"/ (29935)
12     *"Test Reliability"/ (19556)
13     medical diagnosis/ or exp diagnosis/ (87220)
14     exp Questionnaires/ or diagnos*.mp. (204034)
15     exp Child Behavior Checklist/ or checklist*.mp. (36951)
16     (tests and testing).mp. [mp=title, abstract, heading word, table of contents, key concepts, original title, tests
& measures] (14581)
17     exp personality measures/ (16615)
18     11 or 12 or 13 or 14 or 15 or 16 or 17 (315522)
19     6 and 10 and 18 (2616)

Ovid Technologies, Inc

Database: Health and Psychosocial Instruments <1985 to April 2013> Searched June 19, 2013
Search Strategy:
--------------------------------------------------------------------------------
1     [ptsd.mp](http://ptsd.mp/). [mp=title, acronym, descriptors, measure descriptors, sample descriptors, abstract, source] (1154)
2     post traumatic [stress.mp](http://stress.mp/). [mp=title, acronym, descriptors, measure descriptors, sample descriptors, abstract,
source] (293)
3     (acute stress disorder or acute stress symptom*).mp. [mp=title, acronym, descriptors, measure descriptors, sample
descriptors, abstract, source] (78)
4     "Posttraumatic Stress Disorder".sd. (493)
5     "Stress Disorders Post Traumatic".sd. (373)
6     "Posttraumatic Distress*".md. (13)
7     "Stress Reactions*".md. (1379)
8     "Psychological Stress*".md. (965)
9     "Stress Psychological*".md. (1151)
10     "Posttraumatic Symptomatology*".md. (13)
11     "Emotional Trauma*".md. (730)
12     "Stress Disorders Post Traumatic*".md. (501)
13     1 or 2 or 3 or 4 or 5 or 6 or 7 or 8 or 9 or 10 or 11 or 12 (3916)
14     (pediatric* or paediatric* or child* or newborn$ or adolescen* or newborn* or congenital* or infan* or preschool*
or pre-school* or teen$ or kindergarden$ or kindergarten* or elementary school$ or nursery school$ or youth$ or baby$ or
babies or neonat$ or schoolchild* or toddler$ or boy or boys or girl* or pubescen* or juvenile* or adolesc*or
pre-pubesc*).mp. or (child* or adolesc* or pediat* or paediat*).jn. (49537)
15     "Adolescent".sd. (9808)
16     "Child".sd. (6137)
17     "Adolescence".sd. (8810)
18     "Childhood".sd. (2035)
19     14 or 15 or 16 or 17 (49537)
20     13 and 19 (1002)

ProQuest Dissertations & Theses Full Text Searched June 19, 2013

Searched for: all(ptsd OR posttraumatic OR post traumatic OR acute stress) AND all(child* OR pediatric* OR paediatric* OR boy OR boys OR girl OR girls OR adolescent* OR juvenile* OR youth* OR pubescen* OR high school* OR teenage* OR toddler* OR preschool* OR pre-school*) AND all(questionnaire* OR method* OR checklist* OR tool* OR test* OR evaluat*) AND all(diagnos*)

Results: 304°

* Duplicates are removed from your search, but included in your result count.

BASE (Bielefeld Academic Search Engine) Searched June 19, 2013

Search restricted to Books, Reports papers, lectures and Theses categories.

("post traumatic" posttraumatic ptsd) and (child children pediatric paediatric boy girl adolescent youth) and (diagnostic diagnosis test checklist questionnaire assessment evaluation)

Result 6


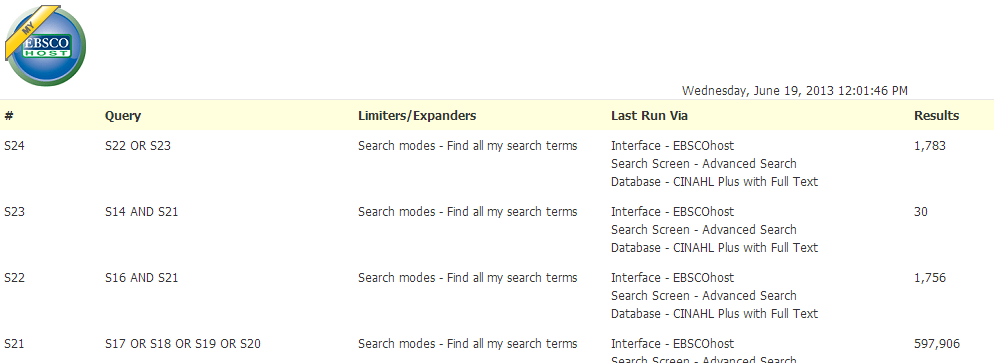

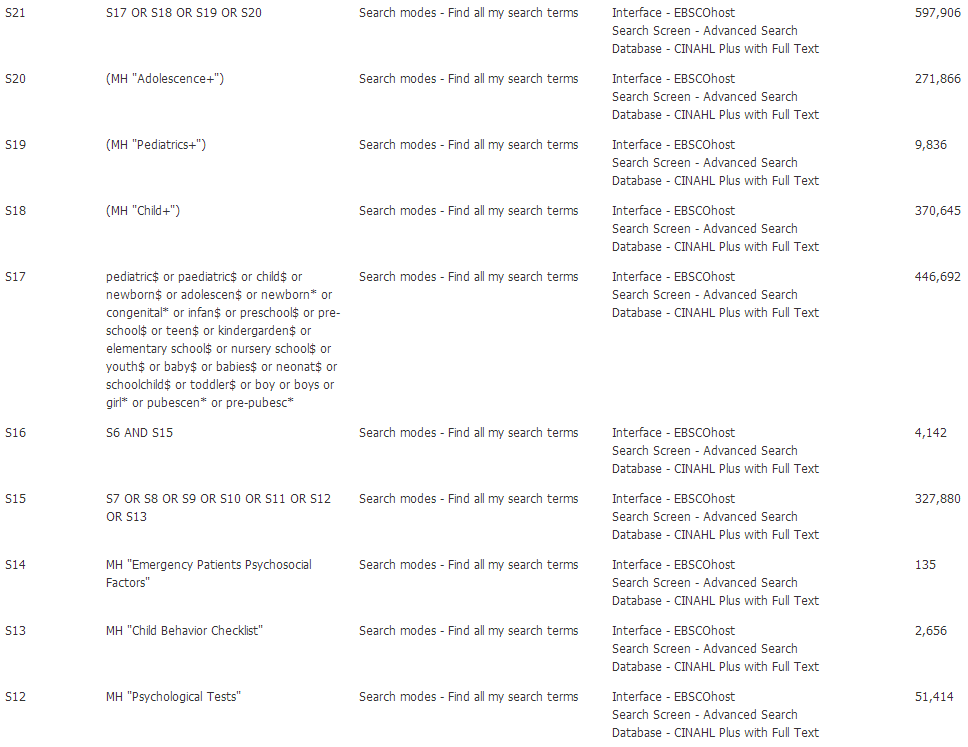

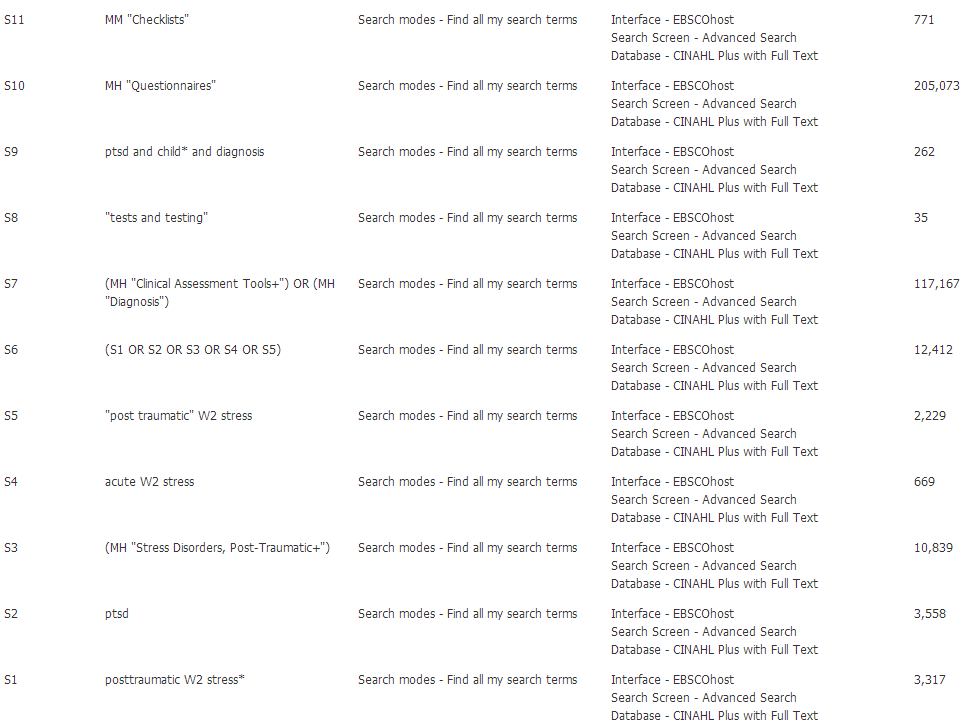


CINAHL Searched June 19, 2013
